# Comparative cytotoxic activity between kaempferol and gallic acid against various cancer cell lines

**DOI:** 10.1016/j.dib.2018.10.121

**Published:** 2018-10-27

**Authors:** Hong Ngoc Thuy Pham, Jennette A. Sakoff, Quan V. Vuong, Michael C. Bowyer, Christopher J. Scarlett

**Affiliations:** a School of Environmental and Life Sciences, Faculty of Science, University of Newcastle, Ourimbah, NSW 2258, Australia; b Faculty of Food Technology, Nha Trang University, No. 2 Nguyen Dinh Chieu Street, Nha Trang City, Khanh Hoa, Vietnam; c Department of Medical Oncology, Calvary Mater Newcastle Hospital, Waratah, NSW 2298, Australia

## Abstract

This data article indicates the *in vitro* cytotoxicity of kaempferol and gallic acid across different cancer cell lines including A2780 (ovarian), H460 (lung), A431 (skin), MIA PaCa-2 (pancreas), Du145 (prostate), HT29 (colon), MCF-7 (breast), BE2-C (neuroblastoma), SJ-G2, U87 and SMA (glioblastoma). The dataset showed that the inhibitory activity of kaempferol was comparatively stronger than gallic acid. Thereby, kaempferol is offered as a potent anticancer agent for further investigation and beneficial as a dietary supplement. The data within this article relates to the research article entitled “Screening phytochemical content, antioxidant, antimicrobial and cytotoxic activities of *Catharanthus roseus* (L.) G. Don stem extract and its fractions” (Pham et al., 2018).

**Specifications table**

**Table:** 

**Subject area**	Biology
**More specific subject area**	Assessment of *in vitro* anticancer properties
**Type of data**	Table, figures
**How data was acquired**	MTT assay
**Data format**	Analyzed
**Experimental factors**	Kaempferol and gallic acid were dissolved in DMSO to generate the desired solutions before being used to treat the cell lines.
**Experimental features**	MTT assay was applied to evaluate the cytotoxic activity of kaempferol and gallic acid against the selected cancer cell lines. Growth inhibition values (GI_50_, concentration that inhibits cell growth by 50%) of these compounds were also determined.
**Data source location**	N/A
**Data accessibility**	Data are presented in this article

**Value of the data**

• The data reveal the inhibitory activity of kaempferol and gallic acid against various cancer cell lines (Figs. 1 and 2).
• It is clearly shown that kaempferol possessed greater *in vitro* anticancer activity than gallic acid, in particular against SMA (Glioblastoma murine), SJ-G2 (Glioblastoma) and A2780 (Ovarian) (Figs. 1 and 2).
• The GI_50_ values confirm the strong cytotoxicity of kaempferol (Table 1).

## 1 Data

**Fig. 1:**
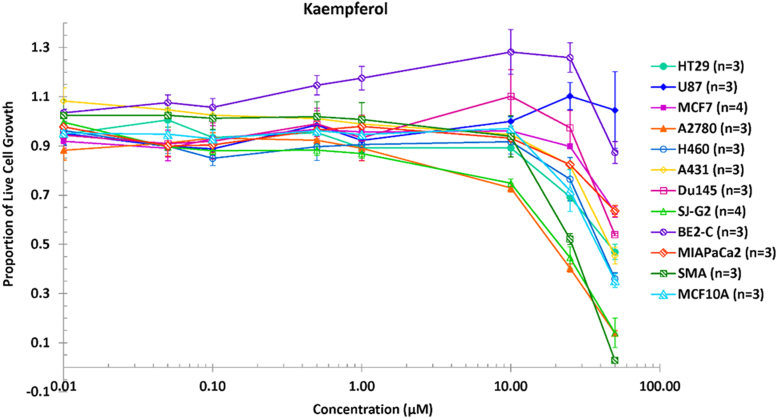
Proportion of live cell growth after treatment with kaempferol at different concentrations.

**Fig. 2:**
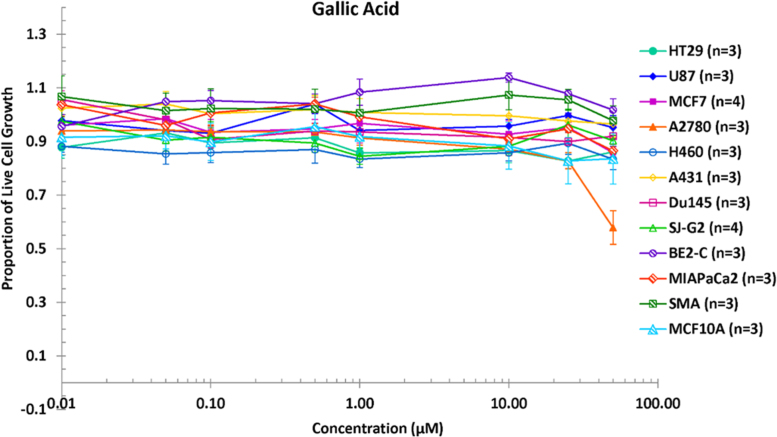
Proportion of live cell growth after treatment with gallic acid at different concentrations.

This article indicates the comparative data of the proportion of live cancer cells after treatment with kaempferol and gallic acid at different concentrations (Figs. 1 and 2), which were found in the *Catharanthus roseus* stem extract and its fractions presented in the research article entitled “Screening phytochemical content, antioxidant, antimicrobial and cytotoxic activities of *C. roseus* (L.) G. Don stem extract and its fractions” [1]. The GI_50_ values of kaempferol and gallic acid across a panel of cancer cell lines, which inhibit the cell growth by 50%, were also determined (Table 1). The lower GI_50_ values indicate stronger growth inhibition.

**Table 1:** Growth inhibition values (GI_50_, concentration that inhibits cell growth by 50%) of kaempferol and gallic acid across a panel of cancer cell lines.

Cell line	Cancer cell types	GI_50_ values (µM)	
		Kaempferol	Gallic acid
A2780	Ovarian	19 ± 0.33	>50
H460	Lung	38 ± 3.30	>50
A431	Skin	46 ± 2.70	>50
MIA PaCa-2	Pancreas	>50	>50
Du145	Prostate	50 ± 0.00	>50
HT29	Colon	45 ± 2.90	>50
MCF-7	Breast	>50	>50
MCF10A	Breast (normal)	37 ± 3.5	>50
BE2-C	Neuroblastoma	>50	>50
SJ-G2	Glioblastoma	22 ± 2.10	>50
U87	Glioblastoma	>50	>50
SMA	Glioblastoma (murine)	26 ± 1.30	>50

The values are means ± standard deviations (*n =* 3).

## 2 Experimental design, materials and methods

### 2.1 Experimental design

Kaempferol and gallic acid were dissolved in DMSO to obtain the stock solutions which were then diluted using relevant media to obtain the working solutions. Tested cells were plated in culture media (100 µL) in a 96-well plate at a density of 2500–4000 cells per well. When cells were at logarithmic growth after 24 h, they were treated with the working solutions of kaempferol and gallic acid to give a final concentration of 50–0.01 µM. After 72 h of incubation, the proportion of live cell growth was determined using the MTT (3-(4,5-dimethylthiazol-2-yl)-2,5-diphenyltetrazolium bromide) assay.

### 2.2 Materials

Kaempferol, gallic acid and dimethyl sulphoxide (DMSO) were purchased from Sigma-Aldrich Co. Human cancer cell lines were obtained from the American Type Culture Collection (ATCC, Manassas, VA, USA). 3-(4,5-dimethylthiazol-2-yl)-2,5-diphenyltetrazolium bromide (MTT) and Dulbecco׳s Modified Eagle׳s Medium (DMEM) were products of Gibco by Life Technologies (Grand Island, NY, USA).

### 2.3 Methods

All tested cancer lines and one non-tumour derived normal breast cell line (MCF10A) were maintained in a humidified atmosphere 5% CO_2_ at 37 °C. All cancer cell lines were cultured in DMEM supplemented with 10% foetal bovine serum, 100 IU/mL penicillin, 100 µg/mL streptomycin and 2 mM l-glutamine. The non-tumour derived MCF10A cells were cultured in DMEM:F12 (1:1) cell culture media, 5% heat inactivated horse serum, supplemented with penicillin (100 IU/mL), streptomycin (100 µg/mL), 20 mM Hepes, l-glutamine (2 mM), epidermal growth factor (20 ng/mL), hydrocortisone (500 ng/mL), cholera toxin (100 ng/mL) and insulin (10 µg/mL). Cytotoxic activity of kaempferol and gallic acid on various cancer cell lines was assessed using MTT assay as described in a previous study [2]. The absorbance values were read at 540 nm to determine the proportion of live cell growth. The growth inhibition values were also determined based on a dose response curve (50–0.01 µM).
